# Autism spectrum disorder and kidney disease

**DOI:** 10.1007/s00467-020-04875-y

**Published:** 2020-12-19

**Authors:** Joanna Clothier, Michael Absoud

**Affiliations:** 1 Department of Paediatric Nephro-urology, Evelina London Children’s Hospital, Westminster Bridge Road, London, SE1 7EH UK; 2 Newcomen Neurodevelopmental Team, Children’s Neurosciences, Evelina London Children’s Hospital, Westminster Bridge Road, London, SE1 7EH UK

**Keywords:** Children, Autism spectrum disorder, Chronic kidney disease, Challenging behaviour, Social stories, Learning disability, Paediatric

## Abstract

Neurodevelopmental impairments have been recognised as a major association of paediatric kidney disease and bladder dysfunction, presenting challenges to clinicians and families to provide reasonable adjustments in order to allow access to investigations and treatments. Autism spectrum disorder (ASD) is a common neurodevelopmental disorder characterised by impairments in social interaction/communication and repetitive sensory-motor behaviours. Mental health, learning and physical co-morbidities are common. There is emerging evidence that ASD and kidney disease have some overlaps with genetic copy number variants and environmental factors contributing to shared pathogenesis. Prevalence rates of ASD in kidney disease are currently not known. A high index of suspicion of underlying ASD is required when a young person presents with communication difficulties, anxiety or behaviour that challenges, which should then trigger referral for a neurodevelopmental and behavioural assessment. We discuss practical approaches for providing care, which include understanding methods of communication and sensory, behavioural and environmental adaptations.

**Supplementary Information:**

The online version contains supplementary material available at 10.1007/s00467-020-04875-y.

## What is autism spectrum disorder?

Autism spectrum disorder (ASD) is a heterogeneous early-onset and lifelong neurodevelopmental disorder which is highly heritable but with as yet no established biomarker [1]. Its dyad of core domain features lies on a dimensional spectrum and are characterised by social communication/interaction deficits and repetitive, unusual sensory-motor behaviours (Table 1). ASD is present in at least 1% of child and adolescent populations worldwide, and the measured prevalence may be increasing [1, 3, 4]. In a recent US active surveillance program that provides estimates of the prevalence of ASD among children aged 8 years, the prevalence of autism was estimated as 18.5 per 1000 (one in 54) [5]. There is a male predominance with male to female ratio estimates of 3–4:1 [6]. The resultant functional impairment ranges from mild to severe. Children and young people often have additional intellectual disability (IQ < 70, in about 30–50%) or meet criteria for an additional language disorder. Individuals and families are primarily treated through education and behavioural services with medication as an aid in some.

**Table 1:** ASD symptoms and signs*

• ASD symptoms must be present in the early developmental period (but might not become fully manifest until social demands exceed limited capacities, or might be masked by learned strategies in later life).	
• Symptoms in the social communication/interaction must include impairments in	
- Social emotional reciprocity (e.g. failure of normal back and forth conversation; or reduced sharing of interests, emotions, or affection)	
- Non-verbal communicative behaviours (e.g. abnormalities in eye contact and body language)	
- Developing, maintaining, and understanding relationships (e.g. difficulties in sharing imaginative play or making friends).	
• Symptoms in the repetitive patterns of behaviour must include impairments in two of	
- Stereotyped or repetitive motor movements, use of objects or speech (e.g. lining up toys, or flipping objects)	
- Insistence on sameness, inflexible adherence to routines or ritualised patterns of verbal and non-verbal behaviour (e.g. extreme distress at small changes, difficulties with transitions or rigid thinking patterns)	
- Highly restricted, fixated interests that are abnormal in intensity or focus (e.g. strong attachment to or preoccupation with unusual objects)	
- Hyper-reactivity or hypo-reactivity to sensory input, or unusual interests in sensory aspects of the environment (e.g. apparent indifference to pain or temperature, or adverse responses to specific sounds or textures).	

*Developed from information in Diagnostic and Statistical Manual of Mental Disorders [2]

The 2013 DSM-5 American Psychiatric Associations Diagnostic and Statistical Manual of Mental Disorders (DSM)-5 criteria recognise that ASD can be accompanied by other conditions, including genetic disorders (e.g. fragile X syndrome), neurodevelopmental disorders (e.g. tic disorders and attention-deficit hyperactivity disorder (ADHD) which occurs in ~ 30% of those with ASD) and psychiatric disorders (e.g. anxiety disorders such as generalised anxiety, separation anxiety and phobias) [2]. Irritability and aggression are also common in ASD (25%) ranging from minor physical aggression in very young children to verbal aggression in adolescents [1]. Other associated problems include sleeping problems (in ~ 1/3), avoidant/restrictive food intake (in ~ 1/2), bladder emptying and constipation [1].

## Genetics of ASD

ASD is highly heritable (64–91%), although environmental factors are also important but relatively understudied [7]. With regards to recurrence risk, ASD occurs in 10–20% of subsequent children after an older child is diagnosed with ASD [8]. ASD models of genetics indicate hundreds of genes implicated and complex inheritance, with contributions from common variants that make small contributions to risk in addition to rare variants (de novo or inherited) that have larger effect sizes, with the different variants converging on common biological pathways [9]. In addition to this heterogeneity, ASD genes have variable penetrance in individuals. It is also increasingly accepted that many variants are associated with other neuropsychiatric phenotypes, particularly intellectual disability, epilepsy and schizophrenia [9]. ASD associated with genetic syndromes is termed ‘syndromic autism’ such as when it arises in the context of mono-genetic disorders including fragile X, and tuberous sclerosis [1, 6]. Current standard of care increasingly includes chromosomal microarray testing for genetic diagnosis particularly when intellectual disability is present. Exome sequencing is then increasingly used as a second line.

## ASD and other medical co-morbidities

It is now recognised that medical conditions are commonly seen in individuals with ASD, particularly when associated with intellectual disability. Initially, these were described in case reports and small case series. A review of population studies performed in 1996 concluded that 25% of individuals with ASD had additional medical conditions [10]. In a US National Survey of Children’s Health (2011–2012) of 1604 children with ASD, 8.6% had epilepsy, and the co-occurrence of epilepsy was associated with increasing child age, female sex and intellectual disability [11].

Population-based studies from around the world have supported the association of ASD with a large number of congenital abnormalities. Registry studies in Denmark identified a highly significant increase in frequency of congenital malformations in the autism population with 11.9% having one or more disorder [12]. A Finnish national birth cohort study with 4441 children with ASD identified abnormalities of eye, central nervous system and specific craniofacial anomalies most strongly associated with ASD. It also found that children with ASD and intellectual disability were more likely to have congenital anomalies [13]. In a population-based longitudinal study in Taiwan of 3440 autistic children born 1997–1999 with 33,391 matched controls, children with ASD were found to have greatly elevated risks of congenital anomalies compared with matched non-autistic peers. The increased risk of medical diseases for children with intellectual disability and autism were approximately 1.6–9 times greater than for isolated autism [14]. A study from Western Australia with siblings and population controls (1980–1995) found that the prevalence of birth defects was significantly higher in ASD cases than in population controls [15].

## Prevalence of ASD in kidney patient populations

A recent paper assessing co-morbidity burden in adults with ASD and intellectual disability (mean age 42.9 years) identified a large range of medical disorders, with 25.4% being found to have chronic kidney disease (CKD) [16].

Neurodevelopmental and mental health impairment are recognised as being long-term morbidities in children with CKD. There are, however, no published papers specifically examining the prevalence of ASD within the paediatric nephrology population and no studies specifically describing the prevalence of kidney disease within the paediatric ASD population. In our own regional referral centre, we retrospectively reviewed a cohort of 224 patients with CKD (estimated GFR < 50ml/min/1.73 m^2^), and we identified 24 as having a confirmed diagnosis of ASD (unpublished data).

Population case-controlled studies are required to investigate whether there is an association between paediatric kidney disease and autism. For example, case-controlled studies recently performed in the congenital heart disease (CHD) population have been conducted due to long-standing association with neurodevelopmental disorders. A nationwide population case-controlled study in Taiwan, and a US case-controlled study in the Military Health System, showed that children with CHD are at increased risk of ASD. The reason for the link between CHD and ASD is unknown, with suggested candidates being shared genetic mutations, acquired brain injury, and patient-specific risk factors such as prematurity, and early-onset epilepsy [17, 18].

## When to consider ASD in children with kidney disease

Neurodevelopmental deficits have long been recognised as a major complication of paediatric CKD. Some of this impairment has been attributed to the possible effects of kidney failure: uraemia, anaemia, hypertension and malnutrition.

The US Chronic Kidney Disease in Children (CKiD) cross-sectional cohort study of children with mild to moderate paediatric CKD (*n* = 368) identified more than double the normative expectation of neurocognitive impairments [19].

In a previous review paper, Gipson et al. recommended the need for prospective neurocognitive evaluation in children with kidney disease to allow early intervention, with the aim of optimising neurodevelopmental, health and educational achievements [20].

In the USA, the American Academy of Pediatrics has for a long time recommended screening for general development and ASD from infancy as part of their autism initiatives. In the USA, all children are screened for ASD at 18 and 24 months of age, along with regular developmental surveillance. When caring for a child with kidney disease, this information can be invaluable in planning for and providing care. However, autism traits are often not apparent until a child is older, particularly in those with good intellectual ability. The presence of emotional and behavioural difficulties such as anxiety or hyperactivity might cause diagnostic overshadowing in these children which can be attributed to the medical condition. Hence, an awareness of the risk of neurodevelopmental differences and possible ASD should prompt referral to neurodevelopmental specialists of any child in kidney clinics deemed outside of normal developmental parameters.

### Genetic syndromes

Kidney disease and ASD can be seen to co-exist in many multisystem genetic disorders (Table 2). Tuberous sclerosis, for example, is a multisystem disorder resulting from mutations in either the *TSC1* or *TSC2* genes, leading to abnormal signalling in the mammalian target of rapamycin (mTOR) pathway. The condition is commonly associated with autism in up to 61% of patients, with the presence of renal angiomyolipomata, cystic kidney disease and hypertension in 80% [21]. Phase 3 everolimus trials, EXIST-1 and EXIST-2, demonstrate that everolimus may reduce angiomyolipoma lesion volume and preserve kidney function in most patients [22]. The EXIST-3 sub-study in Japan found that adjunctive everolimus treatment improved seizure frequency and had a favourable trend towards improvement in ASD symptoms, although these results have not been replicated [23].

**Table 2:** Genetic syndromes that associate kidney conditions and ASD

Kidney disorder group	Condition and genetics
Congenital anomalies of kidneys and urinary tract	Bardel–Biedl syndrome *BBS*
	Brachio-oto-renal syndrome *EYA1*
	CHARGE *CHD7*
	Cornelia de Lange *NIPBL*
	Di George syndrome 22q11.2
	Down syndrome *Trisomy 21*
	FOXP1 syndrome *FOXP1*
	Fragile X *FMR1*
	Fraser syndrome *GRIP1*
	Gabriele-de-Vries syndrome *YY1*
	HDR syndrome *GATA3*
	Jacobsen syndrome *ETS1*
	Kleefstra syndrome *EHMT1*
	Phelan-McDermid syndrome 22q13.3 including *SHANK3*
	Rubinstein Taybi syndrome *CREBBP*
	Smith Lemi-Opitz *DHCR7*
	Smith Magenis syndrome *RAI1*
	Sotos syndrome *NSD1*
	Williams syndrome 7q11.23
	Wolf-Hirshhorn syndrome 4p-
Tubular disease	Familial hyperkalemic hypertension *CUL3* WNK kinases implicated in ASD
	Pseudohypoaldosteronism, type 1 *SLC12A*
	Renal tubular acidosis *CA2*
	Hyperaldosteronism *CACNA1D*
	Lowe syndrome *OCRL*
Cystic disease	RCAD (HNF1beta nephropathy (HNF1B) (if17q12 deletion encompassing *HNF1b)*
	Tuberous sclerosis complex *TSC1, TSC2*
	Nephronophthisis *NPHP1, NPHP6*
	Orofaciodigital syndrome *OFD1*
Cancer	PTEN hamartoma tumour syndrome *PTEN*
	WAGR *PRRG4*
Other	Rett syndrome *MECP2*
	Wilson’s disease *ATP7B*
	Neurofibromatosis *NF1*

### Copy number variants

The CKiD population was examined for any genetic alteration and identified a higher burden of large (> 400 kilobase pairs) gene-disrupting copy number variants (CNVs—a structural variation of the genome involving a duplication or deletion) in children with CKD compared with controls. CNVs were found in 7.4% of the CKiD population and across a range of kidney diagnoses. Most of those found to have pathogenic CNVs had significant neurodevelopmental and psychiatric impairment (lower IQ, depression/anxiety and executive function). The majority of the pathogenic imbalances detected had known associations with developmental delay, intellectual disability and/or seizure disorders, suggesting that genetic abnormalities may affect both the kidney and the neurocognitive function [24–26].

It remains poorly understood how these diverse sets of genes relate to the underlying molecular mechanisms.

Genetic investigations using microarrays have now identified over 200 recurrent pathogenic gene-disrupting CNVs. These often encompass more than one gene, and there has been found to be significant overlap between kidney and neuropsychiatric disorders, hence demonstrating some shared pathogenesis [27].

17q12 and 16q24.2 deletions are two examples of where renal conditions and ASD can co-exist due to the deletion encompassing more genetic material than the gene known to cause kidney disease. Heterozygous mutations of *HNF1B* gene are the commonest-known monogenic cause of developmental kidney disease (RCAD syndrome), accounting for around one-third of hyper-echogenic cystic kidneys in children. The first report of an association with ASD described 3 out of 53 children with cystic or hyper-echogenic kidneys and heterozygous 17q12 region deletion encompassing hepatocyte nuclear factor-1beta (*HNF1B*) [28].

Subsequently, 17q12 deletions encompassing *HNF1B*, but not *HNF1B* intragenic mutations, have been found to be associated with neurodevelopmental disorders; hence, the *HNF1B* gene is not involved in the neurodevelopmental phenotype [29]. But 17q12 deletions encompassing hepatocyte nuclear factor-1beta (*HNF1B*) have been found to be associated with a 14-fold increased risk of suffering with ASD or schizophrenia.

Another example includes 16q24.1-2 deletions. Deletions at 16q24.1 involving the gene *FOXF1* have been found to be associated with congenital kidney malformations. At 16q24.3, deletions involving the gene *ANKRDII* have been found to be associated with ASD and intellectual disability. 16q24.2 deletions have been found to be associated with ASD, intellectual disability and congenital kidney malformation (bilateral hydronephrosis) [30].

### Same gene associated with congenital abnormalities of the kidney and ASD

There is one gene described in the literature which associates a congenital kidney condition with ASD. The *TSHZ3* heterozygous gene deletion at 19q12-13.11 is found to have clinical features of neurodevelopmental disorders (autistic traits), speech delay, intellectual disability as well as renal tract abnormalities (pyelo-calyceal dilatation, VUR, nephrolithiasis). *TSHZ3* is essential for the development of smooth muscle in wall of ureter [31]. *TSHZ3* is also a critical neurodevelopmental regulator in the cortex and cortico-striatal circuitry during pre and postnatal development [32].

### Associated conditions increasing risk of kidney disease and ASD

Children with ASD have higher rates of bladder and bowel dysfunction. They are also reported to have increased rates of nocturnal enuresis and daytime urinary incontinence compared with controls. They often take longer to achieve urinary continence and present with urinary symptoms of urgency and voiding postponement [33].

Kidney conditions and ASD may also be seen in children born prematurely. There is an increased frequency of prematurity in infants with kidney disease, often associated with increased frequency of central nervous system injuries and later developmental impairments. There is a 2–3-fold increase in ASD in childhood to mid-adulthood in infants born preterm [34].

## Providing care for young people with kidney disease and ASD

A diagnosis of ASD can particularly impact upon the delivery of clinical care, but it is also important to consider that intellectual disability occurs in around one-third of these young people. Children with autism and intellectual disability would usually have had their diagnosis before the age of 5 years, and hence have access to specialist educational services and therapies. Health staff should actively engage with education via the parents to ascertain individualised management techniques that are effective during times of distress (e.g. irritability, self-injurious behaviours).

On the other end of the spectrum, as already highlighted, some children with good intellectual ability have later diagnoses of ASD, and may present after onset of their kidney disease. This usually occurs in the context of emotional difficulties and can be overshadowed by the stress caused by managing the kidney disease. Females and those with advanced language are particularly prone to missed or late diagnoses. Often, as social and academic demands increase with age, the ASD impairments become more obvious in this population, leading to failure in academic attainments, mental health difficulties and reduced adaptive functioning which is not explained by the average IQ.

Young people with ASD hence have a diverse range of differences which need to be understood and acknowledged, with reasonable environmental adjustments provided for optimal individualized care that leads to treatment success [35]. Adjustments need to be tailored to the individual’s own strengths and weaknesses. Case example is shown in supplementary material.

It is recognized that the number of hospital admissions for young people with autism is increasing [36].

It is recognized that individuals with ASD have an increased burden of unmet health needs compared with the general population. Hospital healthcare professionals often express anxiety themselves, and fear for patient safety, when looking after young people with ASD, as they often do not feel adequately prepared to care for children with ASD in the hospital setting [37]. Up to 50% of parents have reported safety concerns for their children with ASD whilst in the hospital [38]. A recent systematic review highlighted that when healthcare workers understand and are responsive to children’s individual needs and their neurodevelopmental disability, they are better placed to adjust care delivery processes to improve care quality and safety during hospitalization [39].

To allow full and safe access to health care for the child with ASD regardless of intellectual ability and co-existing diagnosis, there needs to be increased awareness and knowledge amongst healthcare professionals. To achieve this, good communication is required between healthcare professionals, the young person and their parents/carers. Improving awareness of the specific special needs of the child among health professionals can minimize the anxiety these children experience, resulting in a more positive encounter for everyone.

Children with ASD have varying levels of verbal and intellectual ability and may have very specific personal needs. They may struggle to understand new and complex information and, due to different communication needs, communicate pain or anxiety differently, leading to distress which children with ASD may manifest as challenging behaviours. Such behaviours cause anxiety to staff and potential distress to other patients.

The medical environment can be very frightening because of their reduced ability to cope with change in their normal routine, unexpected events and problems understanding what is going on around them. Long periods of time waiting in unfamiliar environments can lead to sensory overload from noise, lights and crowded, busy waiting rooms. The need to undergo investigations and treatment with unfamiliar people, and the process of examination, monitoring and investigations can be particularly challenging to those with hypersensitivity to touch and requirement for personal space.

The hospital environment can lead to the feeling of being overwhelmed and to anxiety, withdrawal, behavioural outburst or repetitive movements such as rocking and hand flapping.

Preparing a child with ASD for hospital admission requires an individualised approach because ASD presents with a wide variety of symptoms [40]. Careful pre-admission preparation in collaboration with caregivers allows exploration of the potential challenges the hospital admission is likely to cause the young person with ASD, and provides information to guide optimal individualized medical care. Detailed admission plans from leaving home to discharge back home, including all required procedures, can be developed with and then shared with the family and key members of staff. Adequately preparing young people with ASD for hospital admission can improve patient and caregivers’ hospital experience by increasing awareness of the child’s communication needs and behaviours [38, 41, 42].

‘Autism-specific care plans’ have been found to improve overall hospital experiences for patients with ASD and improve staff attention to ASD-specific needs [43]. Many hospitals now have pre-admission checklists for patients with ASD, and parents may carry ASD Health Passports.

## Suggested approach to providing care for a young person with suspected or confirmed ASD

1. Neurodevelopmental and behaviour assessment

For the child in order to understand the IQ, language and functional skills of the child, including strengths, weaknesses and any co-existing mental health needs. Understanding the child’s functional age will assist in helping healthcare professionals understand the function of behaviours, and help tailor information delivery at an appropriate developmental communication level. Identifying additional neurodevelopmental and mental health diagnoses will highlight any additional treatable needs (such as anxiety, and ADHD). Assessment needs to be tailored to the individual and their families or carers, in order to enable them to get the right intervention and support from education, health services and voluntary organisations.

2. Understanding methods of communication

Young people with autism may be verbal or non-verbal. Effective communication between the healthcare team and the young person is required prior to delivering treatment (see Table 3 Examples of questions to help staff communicate with a young person with ASD). It is therefore important to understand how the child communicates. Non-verbal children may use sign language, communication tools using visual supports via an electronic device and/or picture exchange communication (PEC) or symbols. These methods of communication need to be used consistently in the hospital setting to avoid the frustration of not being able to communicate needs and anxieties.

**Table 3:** Examples of questions to help staff communicate with a young person with ASD*

• Can they ask you questions?	
• Do the questions need to be short and very specific?	
• Do they need to write things down for you?	
• Would you prefer pictures or symbols?	
• Will it help if they point at things or demonstrate things?	
• Whichever way they communicate with you, will you need a lot of time to think about the question before you can answer it?	
• Do you need extra time when asked questions?	
• Should they ask your carer to help explain things to you?	
• Do you experience pain?	
• What do you do when you experience pain?	
• If you don’t experience pain, how do you know when you are unwell or ill?	

*Created using information from the National Autistic Society website [44]

Those communicating verbally may require simple matter-of-fact language broken down into shorter sequences. Often, direct requests are required to avoid misunderstanding from literal interpretation of speech.

It is important to understand how the young person learns in order to be able to effectively share new information with them. Some children will understand visually presented information better than verbal explanation, while others prefer written information. It may be necessary to act out an intervention or use site-specific photos or pictures in books or communication tools, ‘first/then’ and ‘do/finished’ approaches, social stories and electronic devices.

3. Understand what makes them feel comfortable

It may be helpful to allow a period of familiarisation with the hospital environment, including the introduction of key healthcare staff, together with visits before admission supported with maps and photo stories.

Consideration should be given to nursing in a single side room so as to be able to provide low lighting and a quiet environment. The child could be encouraged to bring in comforting elements from home such as favourite toys, films, fiddle toys, weighted blankets and ear defenders in order to allow them to create a more familiar environment and personal space. Consider adjustments to hospital clothing and identification bands to make the child more accepting of them.

Children with ASD often have a need for sameness, with reduced tolerance of uncertainty. Routines are very different in hospital, so consider developing hospital timetables to reduce anxiety associated with lack of predictability and structure. Communication of time can be achieved with a schedule board, clocks and timers. Where possible, making adjustments to theatre lists by making the child first on the list in order to minimise waiting times can be helpful.

It is important to understand the young person’s sensitivities to auditory, visual and tactile stimuli. Clinical examination is often extremely difficult and anxiety provoking for the patient. Explanation and warning must be given before getting close or touching for physical examinations, investigations and treatment. The young person may struggle to tolerate monitoring equipment such as ECG leads, pulse oximetry, venipuncture and blood pressure cuffs. Explain each step as it occurs, and allow time for the child to explore the equipment or demonstrate it on a trusted adult. During examination, allow access to distractions such as music, puzzles and videos.

4. Rewards (motivators)

It is important to understand what motivates the young person to co-operate. Hospital challenges can be made easier by using good communication and breaking down tasks into smaller steps so that rewards can be easily achieved.

5. Challenging behaviours

Young people with ASD may display behaviours that challenge when they are distressed. It is important to know and understand these behaviours (see Table 4 Examples of ascertaining causes of distress). What are the antecedents to the outburst (e.g. noise, touch) or rigid dislikes and what can be done to diffuse the situation? Parents and carers always have a key role in trying to get co-operation from children, as they know best their child’s specific sensitivities, their usual mode of communication and the sort of rewards that generally produce the desired behaviour. The aim should be to anticipate and pre-empt prior to the outburst occurring.

**Table 4:** Examples of ascertaining causes of distress*

• Do you dislike people getting too close to you?	
• Do you find physical touch unpleasant or distressing?	
• Do you find the smell or feel of rubber gloves unpleasant or distressing?	
• Some of the equipment will be hard and cold—will this be a problem for you?	
• Do you dislike bright lights, especially if they are shining in your eyes?	
• Do you dislike tight things such as blood pressure cuffs?	
• Do you dislike having your blood taken?	
• Will you find it difficult being in an enclosed space, such as a scanner?	
• Do you have difficulty swallowing tablets?	

*Created using information from the National Autistic Society website [44]

Staff can help reduce anxiety by ignoring unwanted behaviour, complimenting cooperative behaviour and dealing with them in a calm and reassuring manner.

Distraction with conversation on topics of their special interest, toys or objects they like, or singing or counting, can be useful in reducing anxiety in a medical setting. When the child is upset, give them space, and aim to identify what triggered the distress.

It is important to consider that many young people with ASD have difficulty expressing pain and other physical symptoms. This can make assessment challenging, and pain can often be communicated through non-specific maladaptive behaviours which can include self-injurious behaviours.

It is useful to learn from previous experience, both positive and negative.

## Conclusion

ASD is a common neurodevelopmental disorder characterised by impairments in social interaction/communication, sensory differences and a need for sameness and reduced tolerance of uncertainty. Mental health (such as anxiety), learning and physical co-morbidities are common. A high index of suspicion of underlying ASD is required when a young person presents with communication difficulties, anxiety or behaviour that challenges, which then trigger referral for a multidisciplinary assessment. Over the last 10 years, there has been an increase in the knowledge of genetic basis of kidney disease, with a highly significant overlap between pathogenic CNVs found in kidney disease and neuropsychiatric disorders. It is important to consider that the identification of monogenic causes of kidney disease can also allow detection, diagnosis and treatment of extra-kidney manifestations earlier, including neurodevelopmental/psychiatric conditions. Population-based studies are required to investigate whether there is an association between paediatric kidney disease and autism. It is likely that children with ASD will be seen regularly in nephrology clinics, hence presenting challenges to clinicians and families to provide reasonable adjustments in order to allow access to investigations and treatments. Clinicians need to have an understanding of ASD and an appreciation of the need for a multi-disciplinary approach in developing individualised medical management plans to optimize outcome. Practical approaches for preparation include understanding methods of communication and sensory, behavioural and environmental adaptations.

## Supplementary Information

ESM 1 (DOCX 15 kb).
