# Supplementary material for: Autism spectrum disorder and kidney disease
Source: Pediatr Nephrol. 2020 Dec 19;36(10):2987–95. doi: 10.1007/s00467-020-04875-y (PMC8445873; doi:10.1007/s00467-020-04875-y)
Supplement: Supplementary file 1 — (DOCX 15 kb). [file 467_2020_4875_MOESM1_ESM.docx]

**Supplementary material**

| **Case Illustration**  A boy with renal cystic dysplasia with bilateral grade IV vesico-ureteric reflux. He was diagnosed with ASD at 3 years of age. From infancy he had required input from the specialist feeding clinic due to sensory feeding difficulties and remained enteral tube-feed dependent. He was non-verbal, requiring speech therapy input and had extensive difficulties with sleep. He had learning disability requiring additional support at school and challenging behaviour.  In the outpatient hospital setting he would struggle with transitioning from one activity to another. He would find having his blood taken challenging and had a history of kicking, punching and head butting when upset and angry about blood taking. This would occur either during the procedure if it was taking too long or once the blood had been taken and was usually directed at the person taking the blood.  Hospital admissions were complicated by pulling out any lines (intravenous lines, urinary catheters) he could see. He also had a history of physically attacking the healthcare staff looking after him.  As his kidney function deteriorated kidney replacement therapy was discussed with the family. It was agreed that the ideal approach would be a pre-emptive living donor kidney transplant.  Together the family and the multidisciplinary team understood adequate preparation would be key in performing a successful transplant and supporting longer term follow up. Preparation for kidney transplant commenced earlier and was more prolonged than for other families and involved his family, carers, the families’ psychologist, his school and the whole of the multidisciplinary team within the hospital (including play specialist, specialist nurses, psychologists, pain specialists, anaesthetists) with involvement from the neurodevelopmental team (doctors, nurse specialists).  Initial work focussed on managing behaviour particularly around unpleasant activities such as blood taking as this was an activity that could not be avoided. To assist with the preparation the psychologist and the hospital play specialist worked together to develop a plan for behaviour. His ‘likes’ and ‘dislikes’ were explored to help develop the approach. The behaviour approach used consistently across all settings hospital, home and school. Routines were developed with immediate reward for desired behaviour and a consistent approach to undesirable behaviour.  To help with understanding hospital procedures a visual timetable with ‘tell, show, do’ pictures were used to aid communication. The approach around unpleasant activities become ‘First we do this’ (the un-preferred activity) followed by the preferred activity, for example use of a game device. Distraction was used for procedures with immediate verbal praise and reward for positive behaviours.  Prior to transplant a behavioural contract was drawn up around blood taking and included the family and hospital phlebotomy team.  Familiarising him and the family with the ward and the dialysis unit was very important in helping reduce anxiety about unfamiliar environments and to gain familiarity with routines in the hospital. Photos of the boy in different areas of the hospital were taken and his own individual transplant book developed. This book was then used both at home and school to prepare him for his transplant. The school developed social stories to be used in the 4 months prior to transplant.  Managing him successfully as an inpatient, particularly his anxiety and pain management following the transplant was of concern. A care contract/support plan was drawn up involving the family, anaesthetist, pain team, autism nurse specialist, allocated nurse, ward sister and chronic kidney disease nurse specialist and consultant. This incorporated, how to get him to theatre and subsequent management after theatre. It was agreed with the family that a pre-med would be given prior to theatre and no intravenous cannulation performed prior to the anaesthetic. After the operation all lines placed would need to be kept covered at all times. The anaesthetic and pain teams agreed management plan for pain/ anxiety and unexpected procedures. A daily review by the pain team with an additional clear medication plan for agitation if the family were not able to manage the behaviour conservatively. The family/carers were asked to be present with him 24-hours a day throughout the inpatient stay to support him. It was agreed the care contract/ support plan would be reviewed regularly.  The support plans were followed and he successfully received a pre-emptive live related kidney transplant from his father at 9 years of age. There were surgical complications following transplantation requiring several trips back to theatre. Unfortunately on one occasion an on-call anaesthetist was involved who failed to follow the agreed plan. This led to difficulties in taking him back to theatre and highlighted the requirement of a clear support plan for this young man.  He is now over 2 years post kidney transplant with stable graft function. He is seen first in the outpatient department and he continues with his phlebotomy plan for blood taking. Planned admissions to the hospital have gone smoothly and have followed the care contracts drawn up for his transplant. He has also requested to go to hospital the last time he felt unwell. |
| --- |
